# Remapping the Chemical Space and the Pharmacological Space of Drugs: What Can We Expect from the Road Ahead?

**DOI:** 10.3390/ph17060742

**Published:** 2024-06-06

**Authors:** Lucas Silva Franco, Bárbara da Silva Mascarenhas de Jesus, Pedro de Sena Murteira Pinheiro, Carlos Alberto Manssour Fraga

**Affiliations:** 1 Laboratório de Avaliação e Síntese de Substâncias Bioativas (LASSBio), Instituto de Ciências Biomédicas, Universidade Federal do Rio de Janeiro, Cidade Universitária, Rio de Janeiro 21941-902, Brazil; silvafrancolucas@gmail.com (L.S.F.); mascarenhas.barbi@gmail.com (B.d.S.M.d.J.); 2 Instituto Nacional de Ciência e Tecnologia de Fármacos e Medicamentos (INCT-INOFAR), Universidade Federal do Rio de Janeiro, Rio de Janeiro 21941-902, Brazil; 3 Programa de Pós-Graduação em Farmacologia e Química Medicinal (PPGFQM), Instituto de Ciências Biomédicas, Universidade Federal do Rio de Janeiro, Cidade Universitária, Rio de Janeiro 21941-902, Brazil

**Keywords:** drug discovery, chemical space, pharmacological space, computational techniques, drug data analysis, network pharmacology

## Abstract

This work examines the current landscape of drug discovery and development, with a particular focus on the chemical and pharmacological spaces. It emphasizes the importance of understanding these spaces to anticipate future trends in drug discovery. The use of cheminformatics and data analysis enabled in silico exploration of these spaces, allowing a perspective of drugs, approved drugs after 2020, and clinical candidates, which were extracted from the newly released ChEMBL34 (March 2024). This perspective on chemical and pharmacological spaces enables the identification of trends and areas to be occupied, thereby creating opportunities for more effective and targeted drug discovery and development strategies in the future.

## 1. Introduction

The concept of chemical space is fundamental in the fields of cheminformatics, medicinal chemistry, natural products, and drug discovery. It can be likened to the cosmos, in that molecules occupy space rather than stars and the whole chemical space refers to the entirety of all possible molecules and known compounds [1]. The total chemical space has been estimated to encompass approximately 10^63^ molecules considering only the presence of atoms C, N, O, or S, with each molecule having a maximum of 30 atoms [2,3,4]. Exploring chemical space is typically accomplished by evaluating chemical libraries. The most comprehensive libraries include large (10^6^–10^7^) stock compound libraries, ultra-large (10^10^–10^15^) on-demand libraries, and generative virtual libraries (10^23^–10^60^) [5].

Approved drugs remain an important source of information in drug discovery. Although not as extensive as the vast chemical libraries that are currently under development, these drugs have undergone rigorous testing and safety assessments, which provide researchers reliable starting points for their investigations. By focusing on these compounds during screening, researchers can directly explore molecules with known pharmacological properties. In addition, repurposing existing drugs for new indications is a faster process than developing new drugs from scratch. If a drug demonstrates promise in screening and disease models, it can potentially be rapidly advanced to clinical trials, thereby accelerating the drug development process [6].

Drugs represent a strategically chosen and diverse subset of chemical space with known pharmacological properties. An example of the effectiveness of libraries based on approved drugs is the Prestwick Chemical Library [7,8]. It is composed of off-patent approved drugs that have been selected to increase the likelihood of identifying high-quality hits. This is achieved by prioritizing high chemical and pharmacological diversity. Prestwick reports that their library has been utilized as a preliminary screening platform for drug discovery initiatives, resulting in prospective candidates for clinical trials. Therefore, libraries based on drugs and constructed upon medicinal chemistry principles and methodologies can enhance the lead-likeness of the hits and augment the success rates of the screening.

In the era of big data, the number of biological activities associated with molecules has increased, making pharmacological space vast and challenging to explore. Concepts related to pharmacological space have been developed to map the boundaries of chemical, target, and disease spaces with the goal of systematizing the search for new drugs. The term “druggable genome” stands out as an approach to map the pharmacological space [9]. It refers to the subset of the approximately 30,000 genes in the human genome that express proteins capable of binding drug-like molecules. This concept is critical to the development of post-genomic research strategies within the pharmaceutical industry. The druggable genome is limited to four types of macromolecules that can be targeted with small-molecule therapeutics: proteins, polysaccharides, lipids, and nucleic acids. However, due to issues of toxicity, selectivity, and the inability to obtain potent compounds against the latter three types, most successful drugs achieve their activity by binding to and modifying the activity of a protein [10,11].

Drugs are the molecules that have been most extensively studied pharmacologically, due to the inherent amount of pharmacological data required to progress through preclinical and clinical studies. In addition, approved drugs are often subject to repurposing studies, which increases the number of biological activities associated with this class of compounds. Studies have identified 893 human and pathogen-derived biomolecules through which 1578 U.S. FDA-approved drugs act. These biomolecules include 667 human genome-derived proteins that are targeted by drugs for human diseases [12].

In the quest to explore chemical and pharmacological spaces, large databases that provide a comprehensive collection of annotated data are of critical importance. Compound libraries and databases are often divided into public and commercial. Examples of public resources that allow searching for compound structures or bioactivities include ChEMBL [13], ZINC [14], and PubMedChem [15]. Commercial libraries such as WuXi’s GalaXi Space (approximately 8 billion compounds), Otava’s CHEMriya (11.8 billion compounds), and Enamine’s REAL Space (36 billion compounds) are examples of large collections that are also diverse and have low overlap (less than 10%) between them [16,17]. These libraries are particularly useful for testing new compounds and providing building blocks for the synthesis of new chemical entities, thus expanding the pharmacological and chemical space.

The vast and complex landscape of drug discovery makes the quest for new drugs challenging. A key aspect of this process involves understanding the chemical space and bioactivities of approved drugs. This study aims to provide a perspective in this domain by exploiting information from the newly released ChEMBL34 (March 2024). ChEMBL is a manually curated database of bioactive molecules with drug-like properties. It brings together chemical, bioactivity, and genomic data. The chemical space of drugs was mapped and compared with recently approved drugs, those approved after 2020, and with clinical candidates. This was followed by an analysis of their bioactivities, which focused on the range of proteins that these drugs interact with in order to determine their target profiles. The aim of this work was to provide an overview of the current state of approved drugs by combining these chemical datasets.

## 2. Results and Discussion

### 2.1. Data Curation

Using the ChEMBL database version 34 (ChEMBL34), three datasets were collected for analysis:

(i) Approved drugs.
(ii) Drugs approved after 2020.
(iii) Drug candidates in clinical development.

The datasets were sampled according to the procedures outlined in the methodology. The objective was to select small-molecule drugs or drug candidates with molecular weights between 100 and 1000 Da. Using these criteria, we were able to analyze key data from the current chemical and pharmacological landscape and identify future trends in drug discovery. The “approved drugs” dataset included 1834 unique drugs, the “approved drugs after 2020” dataset comprised 87 unique drugs, and the “drug candidates in clinical phase” dataset contained 685 small molecules in clinical development.

### 2.2. Chemical Space Analysis

**Figure 1:**
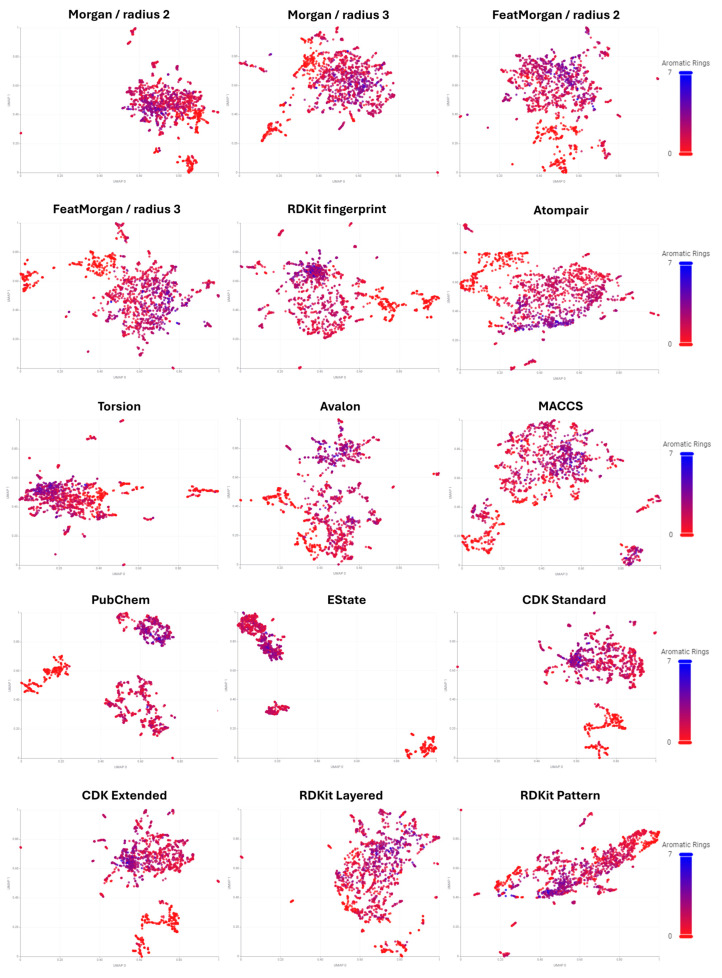
Plot of UMAP embeddings of fingerprints available in RDKit and CDK packages. Drugs are colored based on aromatic ring count.

Chemical space of drugs and drug candidates was determined using cheminformatic tools. Molecules were transformed into chemical fingerprints from RDKit (v. 4.9.1) and CDK (v. 1.5.6) software available on the KNIME platform (v. 5.2.3). Representing molecules as fingerprints furnishes high-dimensional vectors that can be projected in two-dimensional space for visualization. The uniform manifold approximation and projection for dimension reduction (UMAP) technique was applied to reduce high-dimensional chemical information expressed in binary code to two dimensions (Figure 1).

UMAP focuses on maintaining both local and global structure of the dataset as extensively as possible. This method is suitable for understanding both the local structure (organization of similar compounds) and global structure (organization of groups of different compounds) of complex datasets. In general, UMAP displays numerous separate, tight clusters that do not follow a discernible pattern. At a superficial level, one cannot extract information from these plots alone. However, analyzing the compounds represented in these plots using chemical descriptors, such as the count of aromatic rings, helps develop an understanding of how different fingerprints yield varied patterns in chemical space [18].

Aromatic rings are known to be important components of drugs due to numerous reasons. These rings present structural stability due to their planar nature and resonance stabilization, ability to make non-covalent intermolecular interactions, such as pi-stacking, and versatility for chemical modifications, serving as versatile scaffold lead optimization [19]. Overall, they are a fundamental component in the field of medicinal chemistry, and in the dataset of the total approved drugs, 81% (1494 molecules) had at least one aromatic ring.

Therefore, various fingerprints were utilized to describe the chemical structures of drugs and analyzed for their correlation with the count of aromatic rings. Figure 1 illustrates the UMAP embedding of 15 different chemical fingerprints used in the study. Several types of fingerprints were examined, including path-based atom pair fingerprints, which analyze paths through the molecular graph by collecting all possible triplets of two atoms and the shortest path connecting them [20]. Substructure-based fingerprints, such as PubChem fingerprints, encode each bit to indicate the presence of a predefined structural moiety in the compound [21]. Circular fingerprints, like extended connectivity fingerprints (ECFPs), break down target molecules into fragments. This process involves initially representing each atom based on properties such as atomic mass or valence, then adding numerical identifiers for neighboring atoms to generate fragment identifiers. This process is repeated several times, and all unique fragments for a given molecule are hashed into a fixed-size vector.

The analysis of aromatic ring count indicated that PubChem substructure-based fingerprints, which encode the presence of specific structural moieties in each bit, are the most effective for separating compounds into distinct groups based on non-aromatic and aromatic compounds. This approach also provided both local and global clustering of chemical structures [21].

**Figure 2:**
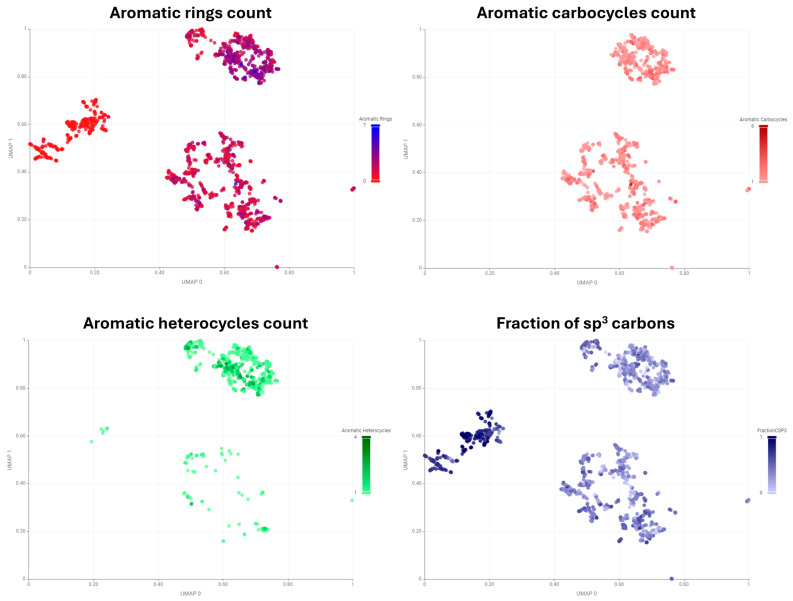
Plot of UMAP embeddings of PubChem fingerprints. Drugs are colored based on aromatic ring count, aromatic carbocycle count, aromatic heterocycle count and fraction of sp^3^ carbons.

Additional PubChem plots serve to reinforce the differences between groups of compounds by comparing the number of aromatic carbocycles, heterocycles, and the fraction of sp^3^ carbons (Figure 2). As anticipated, the molecules situated to the left of the x-axis are not included in the aromatic carbocycle count plot, and few are present in the aromatic heterocycle count plot. Moreover, the fraction of the sp^3^ carbon plot indicates that the molecules in the left part of the 2D plot exhibit a higher sp^3^ character, suggesting that this group contains a greater proportion of aliphatic compounds.

This observation is confirmed by the analysis of this dataset employing the clustering algorithm k-medoids. Using k-medoids for clustering a dataset is interesting, particularly due to its ability to select a representative sample of the data. In addition, k-medoids select actual data points (medoids) as the center of clusters. This makes k-medoids more robust to outliers and noise, as the chosen medoids represent typical data points within the clusters. Since the medoids are actual data points, the resulting clusters are easier to interpret and analyze. Users can directly examine the medoid of each cluster to understand the central characteristics of the cluster.

For selecting the number of clusters, the silhouette score was selected as a metric, given that it measures the cohesion (how close points in a cluster are) and separation (how distinct a cluster is from others). A higher silhouette score indicates better-defined clusters. Using this metric helps in determining the optimal number of clusters by balancing internal cohesion and external separation, leading to more meaningful and interpretable clustering results. This approach avoids the overfitting and underfitting issues commonly associated with arbitrary cluster selection [22].

**Figure 3:**
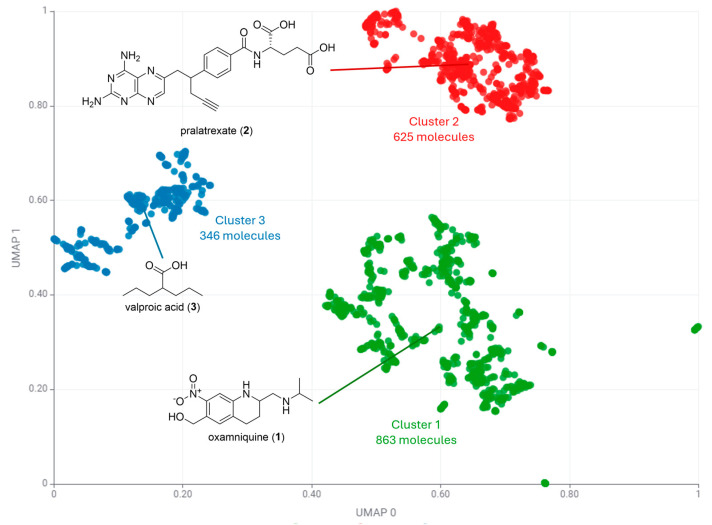
Clustering algorithm k-medoids applied to UMAP embeddings of PubChem fingerprints. Chemical structures of medoids of each cluster are represented.

A silhouette score based on k-medoids applied to the PubChem-based UMAP embedding yielded the highest value for k = 3 (0.71, Figure S1, Supplementary Information). Figure 3 highlights these three clusters and is in accordance with the data illustrated in Figure 1. It can be observed that Cluster 1 has a low prevalence of aromatic heterocycles, but a high contribution of aromatic carbocycles. Cluster 2 is distinguished by a high prevalence of aromatic heterocycles, while Cluster 3 is characterized by a high fraction of sp^3^.

Furthermore, the centroids associated with these clusters support this observation (Figure 3). Cluster 1 (863 molecules) has as its centroid oxamniquine (**1**), an anthelmintic drug used primarily in the treatment of schistosomiasis. Oxamniquine has a nitro-aromatic carbocycle fused to a piperidine ring. Cluster 2 (625 molecules) has as its centroid pralatrexate (**2**), a chemotherapy drug that inhibits dihydrofolate reductase. Pralatrexate (**2**) is a folate and has the aromatic heterocycle 2,4-diaminopteridine in its structure. Cluster 3 (346 molecules) has as its centroid valproic acid (**3**), a drug used to treat epilepsy and other diseases. This compound is an aliphatic carboxylic acid with a calculated fraction of sp^3^ carbons of 0.88.

A second round of clustering was employed to understand the chemical diversity of the groups highlighted in Figure 3 and to further verify the trends suggested in Figure 2. This additional clustering can provide deeper insights and uncover subclusters, as initial clustering might group broad clusters that still contain diverse subgroups. Therefore, a second clustering can reveal finer substructures within these groups, offering more detailed insights. Given that approved drugs, expressed as fingerprints, represent a multidimensional and complex dataset, initial clustering might miss intricate patterns. A secondary clustering can adapt to these complexities, identifying meaningful patterns missed in the first pass. Additionally, it reveals different aspects of the data that a single clustering pass might overlook. Another important aspect is that a second round of clustering helps validate the hypotheses generated in the previous clustering and the observations of descriptors described in Figure 1. If subclustering shows consistent patterns, it reinforces the validity of the initial clustering.

To achieve the second round of clustering, the silhouette score was applied with a maximum k = 100 (Figures S2–S4, Supplementary Information). This value was selected because the structure of the clusters had several subclusters. For visualization purposes, two aspects of the silhouette score were considered. First, an analysis of the best silhouette score for k ≤ 5 was performed to gain a global overview of each cluster. Second, the best silhouette score for k ≤ 100 was analyzed to obtain information on the overall diversity of the clusters (Table 1). In the case of Cluster 3, the best silhouette score was for k = 2. Therefore, the second-best score was selected for k ≤ 100 analysis.

**Figure 4:**
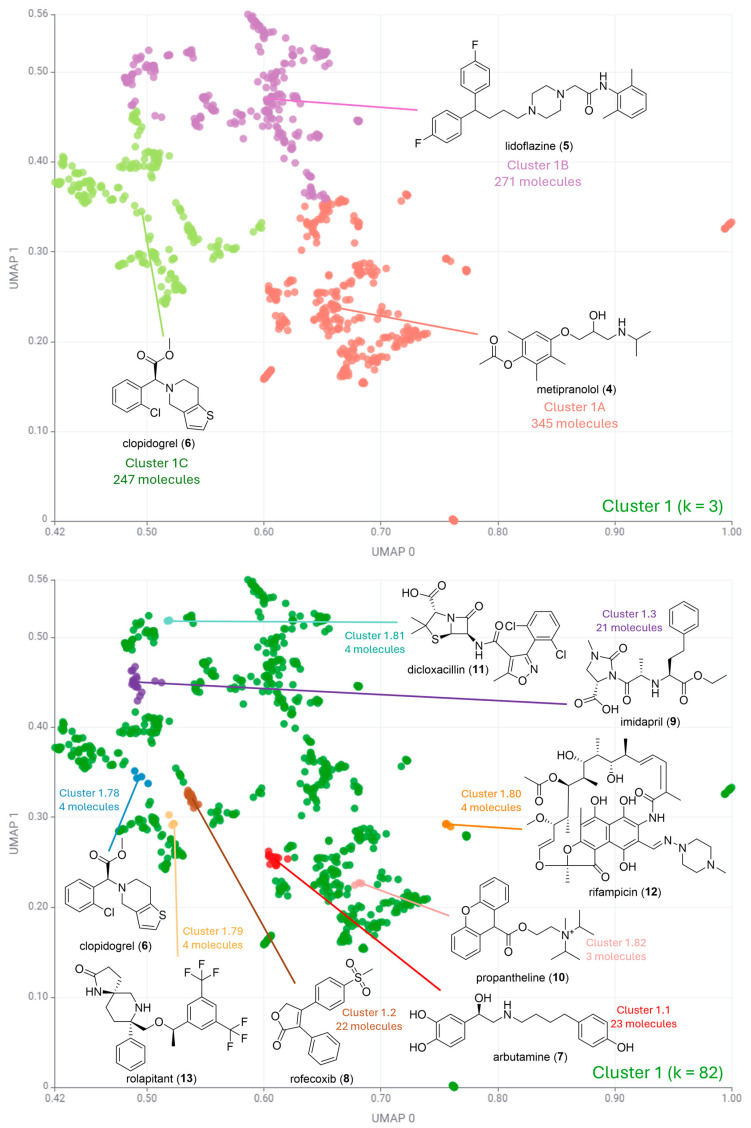
Clustering algorithm k-medoids applied to Cluster 1. For k = 3 (**top**) all medoids are depicted in the plot. For k = 82 (**bottom**), the most and least populated clusters were selected for medoid illustration.

**Figure 5:**
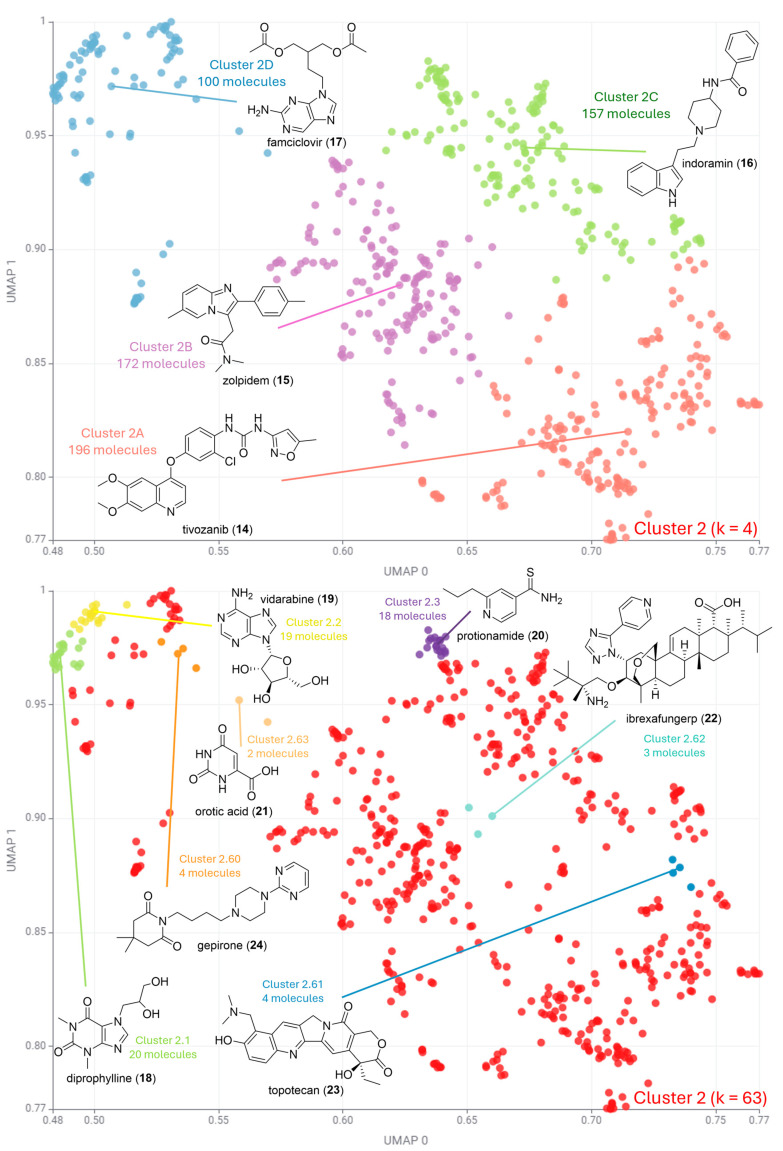
Clustering algorithm k-medoids applied to Cluster 2. For k = 4 (**top**), all medoids are depicted in the plot. For k = 63 (**bottom**), the most and least populated clusters were selected for medoid illustration.

**Figure 6:**
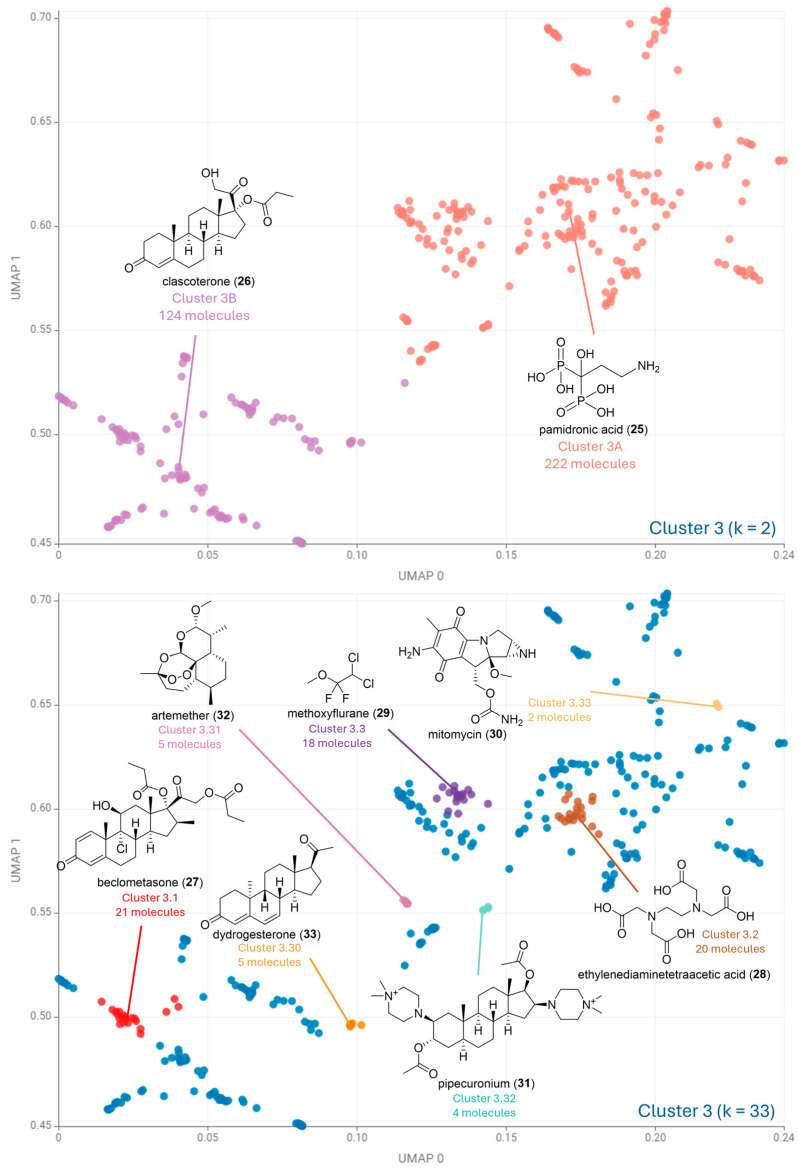
Clustering algorithm k-medoids applied to Cluster 3. For k = 2 (**top**), all medoids are depicted in the plot. For k = 33 (**bottom**), the most and least populated clusters were selected for medoid illustration.

Figure 4, Figure 5 and Figure 6 illustrate the second round of clustering. For cases where k ≤ 5, all medoids are depicted in the plot. For k ≤ 100, the three most and three least populated clusters were selected for medoid illustration. In cases where more than one cluster had the same number of data points, all such clusters were selected for illustration. This occurred particularly in little-populated clusters, where more than three molecules are often illustrated.

Figure 4 shows that the most populated Cluster 1 indeed has predominantly compounds with aromatic carbocycles. Cluster 1 was divided into three clusters, with metipranolol (**4**) as the medoid of the most populated subcluster (Cluster 1A, 345 molecules out of 863). Metipranolol (**4**) is a non-selective beta-adrenergic receptor blocker used to treat glaucoma and cardiovascular diseases, and has a core structure of a phenoxypropanolamine, which is typical for many beta-blockers [23]. The second-most populated subcluster has lidoflazine (**5**) as its medoid (Cluster 1B, 271 molecules). Lidoflazine (**5**) is a calcium channel blocker primarily used as an anti-anginal and anti-arrhythmic agent. This drug has three phenyl rings in its structure, corroborating previous data [24]. The third-most populated cluster (Cluster 1C, 247 molecules) has clopidogrel (**6**) as medoid. Clopidogrel (**6**) is an antiplatelet pro-drug that prevents blood clots by inhibiting platelet aggregation [25]. It has thiophene heterocycle in its structure, suggesting the contribution of aromatic heterocycles to this group, as highlighted in Figure 2.

The highest silhouette score for Cluster 1 corresponds to a value of k = 82 (Figure 4), suggesting a high chemical diversity in this group. The most populated clusters had arbutamine (**7**), rofecoxib (**8**), and imidapril (**9**) as medoids, with cluster populations of 23, 22, and 21 molecules, respectively. Drugs **7**–**9** have phenyl rings, and none has aromatic heterocycles in its structure. Interestingly, both albutamine (**7**) and imidapril (**9**) are used to treat cardiovascular diseases. Albutamine (**7**) is a synthetic sympathomimetic amine that acts as a beta-adrenergic agonist, and imidapril (**9**) is an angiotensin-converting enzyme (ACE) inhibitor.

The least populated clusters (Figure 4) include more complex molecules in terms of the number of chiral centers such as rolapitant (**13**), a selective and long-acting neurokinin 1 (NK1) receptor antagonist that prevents both acute and delayed nausea and vomiting associated with chemotherapy. Natural products or derivatives, such as dicloxacillin (**11**) and rifampicin (**12**), are also included in this fraction. Dicloxacillin (**11**) is a beta-lactam antibiotic derived from the natural product penicillin [26], and rifampicin (**12**) is a semi-synthetic antibiotic from the class of rifamycins [27].

Figure 5 shows that Cluster 2 has predominantly compounds with aromatic heterocycles, with all medoids having at least one of such rings in their structure. Cluster 2 was divided into four clusters, with tivozanib (**14**) as the medoid of the most populated subcluster (Cluster 2A, 196 molecules out of 625). Tivozanib (**14**) is a quinoline-derived VEGF receptor tyrosine kinase inhibitor that blocks angiogenesis in tumors [28]. In addition, **14** has an isoxazole ring in its structure. The second-most populated subcluster has zolpidem (**15**) as its medoid (Cluster 2B, 172 molecules). Zolpidem (**15**) is an imidazopyridine derivative with hypnotic activity by enhancing GABA activity, inducing sedation [29]. The third-most populated cluster (Cluster 2C, 157 molecules) had indoramin (**16**) as medoid. Indoramin (**16**) is an alpha-1 adrenergic receptor antagonist indole derivative. Its mechanism of action leads to vasodilation and decreased blood pressure. The least populated cluster (Cluster 2D, 157 molecules) in this step has famciclovir (**17**) as its medoid. Famciclovir (**17**) is a nitrogenous base analogue prodrug. It is converted to penciclovir triphosphate in infected cells, which inhibits viral DNA polymerase, preventing viral DNA synthesis.

The highest silhouette score for Cluster 2 corresponds to a value of k = 63 (Figure 5). The most populated clusters had diprophylline (**18**), vidarabine (**19**), and protionamide (**20**) as medoids, with cluster populations of 20, 19, and 18 molecules, respectively. Notably, diprophylline (**18**) are vidarabine (**19**) nitrogenous base analogues. Diprophylline (**18**) is a xanthine derivative that relaxes bronchial smooth muscle and stimulates the respiratory center in the brain [30], and vidarabine (**19**) inhibits viral DNA polymerase by being incorporated into viral DNA, causing chain termination. Even though it was in the global structure of Cluster 2, this chemical class was the least populated group (Cluster 2D, 157 molecules). Represented by famciclovir (**17**), it had two of the most populated clusters in terms of diversity when analyzing the maximum silhouette score. This result suggests that nucleoside analogues are a coherent class of drugs, with numerous compounds belonging to this chemical class.

The least populated clusters (Figure 5) include natural product-derived drugs such as ibrexafungerp (**22**) and topotecan (**23**). Ibrexafungerp (**22**) inhibits glucan synthase, impairing fungal cell wall synthesis. It is a semi-synthetic triterpenoid derivative of the natural product enfumafungin [31]. Despite being a triterpenoid derivative, it has a 1,2,4-triazole ring linked to a pyridine, which supports its presence in Cluster 2. Topotecan (**23**) is an anticancer derivative of the natural product camptothecin. Interestingly, **23** also has a quinoline ring in its structure, similar to tivozanib (**14**), which was the main representative in the global analysis of the cluster (Cluster 2A). Topotecan (**23**) also belongs to Cluster 2A, and its anticancer activity is due to its ability to inhibit topoisomerase I, preventing DNA replication and transcription in cancer cells [32].

Figure 6 shows that Cluster 3 has predominantly compounds with a high fraction of sp^3^ carbon, where none of the medoid compounds has aromatic rings. Cluster 3 was divided into two subclusters in the global analysis, with pamidronic acid (**25**) as the medoid of the most populated subcluster (Cluster 3A, 222 molecules out of 346) and clacosterone (**26**) as the medoid of Cluster 3B (124 molecules). Pamidronic acid (**25**) is a bisphosphonate used to prevent osteoporosis [33], and clacosterone (**26**) is a steroidal antiandrogen that competes with dihydrotestosterone, reducing sebum production in acne treatment [34].

The highest silhouette score for Cluster 2 corresponds to a value of k = 33 (Figure 6). This suggests a reduced diversity when compared to Clusters 1 and 2. This is supported by the presence of several steroid analogues as medoids in this analysis. The most populated cluster (21 molecules) had the corticosteroid beclomethasone (**27**) as medoid [35,36]. In addition, the little-populated clusters 3.30 and 3.32 had dydrogesterone (**33**) and pipecuronium (**31**) as medoids. Dydrogesterone (**33**) is a synthetic progestogen that mimics the effects of natural progesterone, regulating the luteal phase and menstrual cycle. Pipecuronium (**31**) is a bisquaternary steroidal drug with non-depolarizing neuromuscular activity used to induce skeletal muscle relaxation during anesthesia [37].

The remaining most populated cluster had ethylenediaminetetraacetic acid (EDTA) (**28**) and methoxyflurane (**29**) as medoids of Cluster 3.2 (20 molecules) and Cluster 3.3 (18 molecules) (Figure 6). Both are acyclic compounds, suggesting the contribution of this feature to some drugs. EDTA (**28**) is used to treat acute and chronic heavy metal toxicity, such as lead poisoning [38], and methoxyflurane (**29**) is an inhalational anesthetic that provides rapid short-term analgesia. The remaining clusters had natural product-derived drugs, such as arthemeter (**32**) and mitomycin (**30**). Artemether (**32**) is a methyl ether derivative of artemisinin, and its endoperoxide bridge generates free radicals through heme-mediated cleavage, damaging the malaria parasite. Mitomycin (**30**) is an aziridine-containing natural product that cross-links DNA, inhibiting DNA synthesis and inducing apoptosis in cancer cells.

**Figure 7:**
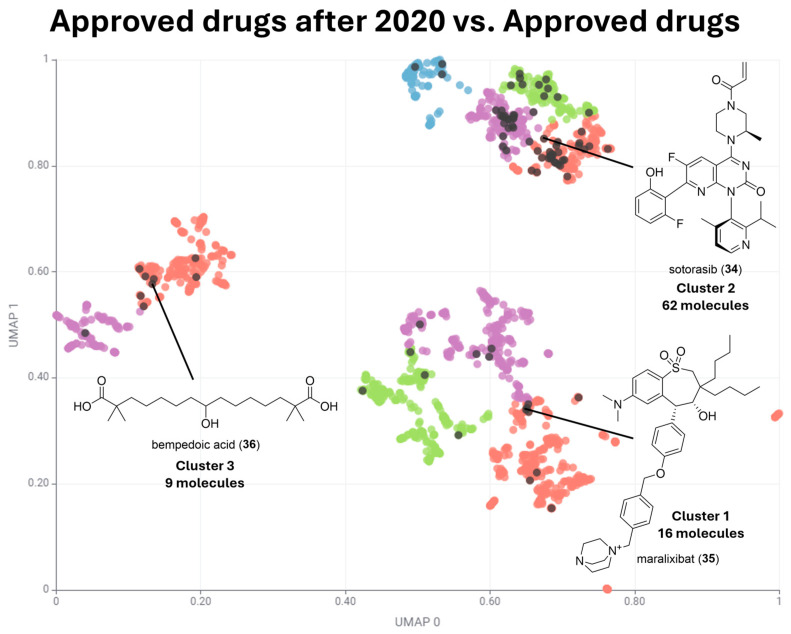
Superposition of chemical space of drugs approved after 2020 and approved drugs. Drugs approved after 2020 are colored in black and approved drugs are colored based on subclusters of Clusters 1, 2, and 3 as described in Figure 4, Figure 5 and Figure 6.

When comparing clustering data exposed in Figure 4, Figure 5 and Figure 6 with drugs approved from 2020 (Figure 7 and Table 2), it can be noted that the most populated cluster shifted from Cluster 1, which has predominantly aromatic carbocyclic compounds, to Cluster 2, which has predominantly aromatic heterocyclic compounds. Cluster 3 remained the least populated cluster.

Figure 7 shows that the most populated Cluster 2 of approved drugs after 2020 has sotorasib (**34**) as the medoid. Sotorasib (**34**) is a targeted cancer therapy that specifically inhibits the KRAS^G12C^ mutation, which is a common driver mutation in various cancers, particularly non-small-cell lung cancer (NSCLC). Sotorasib (**34**) is a pyrido[2,3-d]pyrimidin-2(1*H*)-one derivative bearing an electrophilic warhead acrylamide, which enables irreversible binding to its target, locking it in an inactive state and thereby inhibiting the downstream signaling pathways that promote tumor cell proliferation and survival [39].

Cluster 1, which has predominantly aromatic carbocycles, had maralixabat (**35**) as its medoid. Maralixabat (**35**) is an inhibitor of the apical sodium-dependent bile acid transporter (ASBT), also known as the ileal bile acid transporter (IBAT), responsible for the reabsorption of bile acids from the ileum back into the liver. This reduction in bile acid levels helps alleviate the symptoms associated with cholestatic liver diseases, such as pruritus [40].

Cluster 3, which has predominantly drugs with a high fraction of sp^3^ carbons, had bempedoic acid (**36**) as its medoid. Bempedoic acid (**36**) is a medication primarily used to lower LDL cholesterol levels in individuals who are unable to achieve target cholesterol levels through diet and statin therapy alone. It works by inhibiting adenosine triphosphate–citrate lyase (ACL), an enzyme involved in the production of cholesterol in the liver [41]. In accordance with the clustering of drugs, which had valproic acid (**3**) as medoid (Figure 3), bempedoic acid (**36**) is an aliphatic pentadecanedioic acid.

The results of the clustering analysis indicate that the use of multiple steps of clustering based on chemical fingerprints can assist in the understanding of the hierarchy of the data, as well as in the visualization of the relationships between the subclusters and the main clusters. By breaking down larger clusters into smaller, more homogeneous subclusters, it is possible to achieve more accurate and specific observations about the approved drugs.

However, it is challenging to capture all the complexities of chemical space in two dimensions, which can result in the loss of substantial information. For example, the analysis of clinical candidates did not yield meaningful insights. All clinical candidates were tightly superimposed onto approved drugs, failing to reveal any new trends (Figure S5, Supplementary Material), in contrast to the comparison with drugs approved after 2020. Low-dimensional embeddings are only as effective as their high-dimensional chemical fingerprints. Despite these limitations, the plots still offer significant insights. Notably, global analysis of clusters often aligns with more detailed subcluster descriptions. The analysis of chemical descriptions, such as aromatic ring count and fraction of sp^3^ carbons, indicated which chemotypes were present in each cluster. Cluster analysis corroborated this feature. Furthermore, drugs with similar clinical indications were clustered together based on chemical similarity.

The shift in cluster density when comparing approved drugs with recently approved drugs is corroborated by the clear interest in developing kinase inhibitors, which are mostly aromatic heterocyclic derivatives. To substantiate this hypothesis and comprehend how drugs are currently being studied beyond their mechanism of action, such as in drug repositioning, the following section will analyze the pharmacological space of drugs.

### 2.3. Pharmacological Space Analysis

Post-genomic analysis of drugs is a crucial area of research in pharmacology. This approach involves studying the effects of drugs on gene expression and protein function. It has become a key component of drug discovery since the completion of the Human Genome Project. It has significantly advanced our understanding of drug actions and interactions within biological systems. It has also been instrumental in identifying novel drug targets and repurposing existing drugs for new indications [42]. These analyses have also raised questions about the traditional “one drug, one target, one disease” paradigm of drug discovery. This is because most drugs on the market appear to act through a multitarget mechanism of action, which has brought to light a new concept known as network pharmacology [9,42,43,44].

Network pharmacology highlighted the importance of analyzing the pharmacological space from a holistic perspective [45]. The seminal works conducted by Hopkins and Groom (2002) [9] and Paolini and coworkers (2006) [46] have shed light on the understanding of the pharmacological space by reporting the relationship between the number of pharmacological targets modulated by drugs. Therefore, analyzing the pharmacological space from a network pharmacology perspective is critical. This approach considers that each chemical entity can modulate multiple pharmacological targets. The datasets of Approved Drugs, Approved Drugs after 2020, and Drug Candidates in Clinical Phase were used to determine the total activity count of each chemical entity against pharmacological targets. This analysis includes assays for single proteins in the CHEMBL34 database. Through the analysis of the total activities count, it was possible to assess the distribution of activities in the current scenario and observe trends to understand what to expect ahead in terms of pharmacological targets that are being pursued.

**Figure 8:**
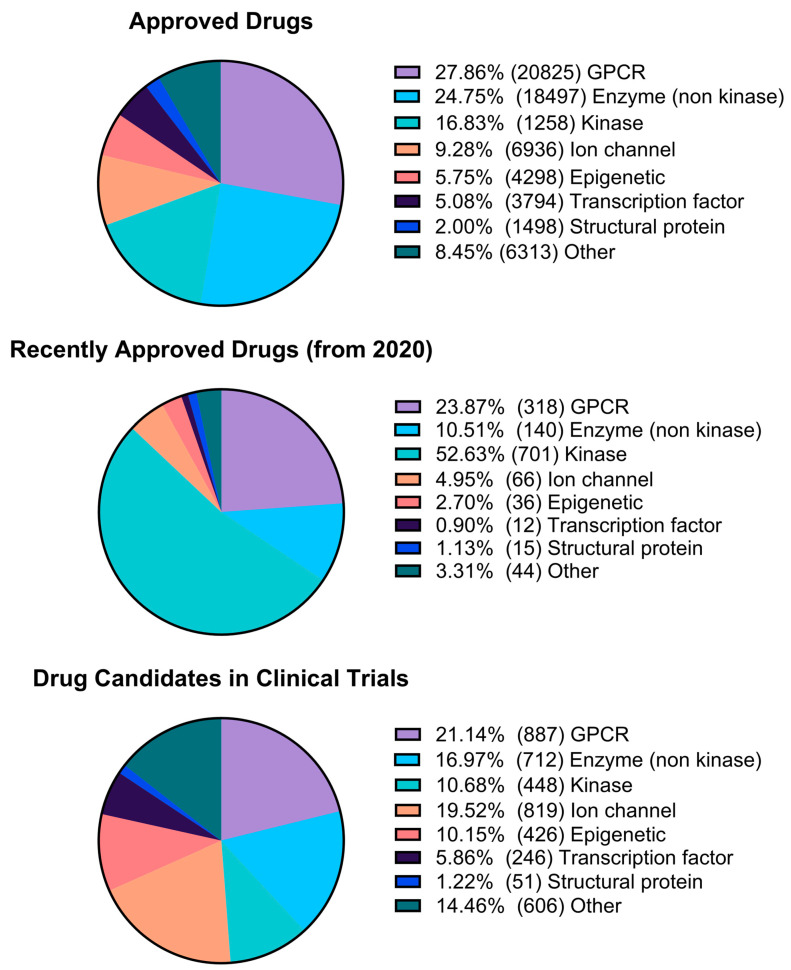
Pharmacological space analysis of the Approved Drugs, Approved Drugs after 2020, and Drug Candidates in Clinical Phase datasets.

**Figure 9:**
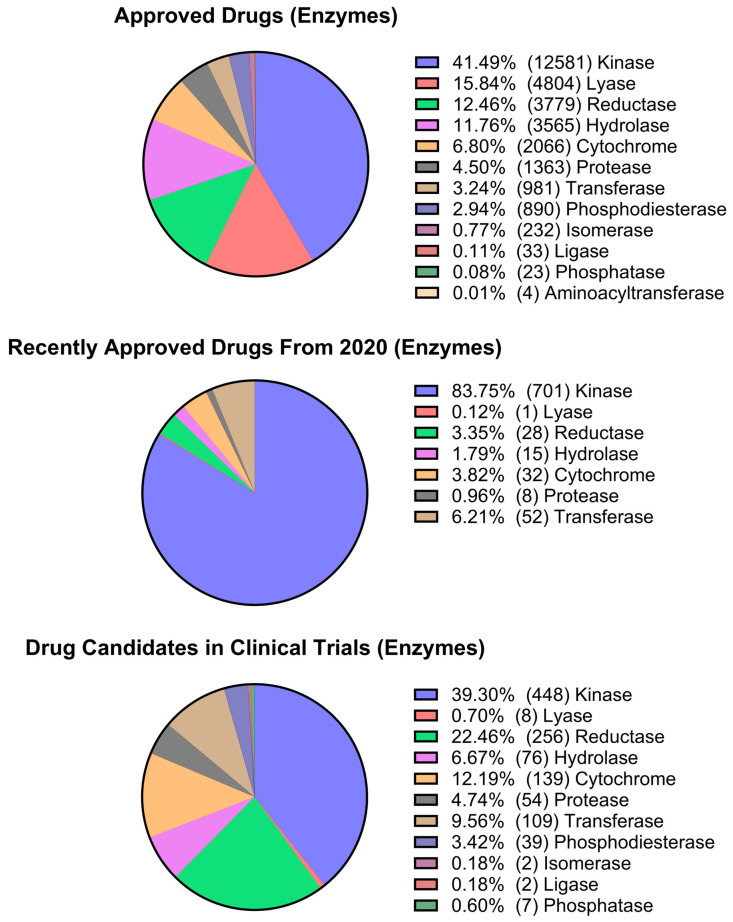
Pharmacological space analysis of enzymes for the Approved Drugs, Approved Drugs after 2020, and Drug Candidates in Clinical Phase datasets.

**Figure 10:**
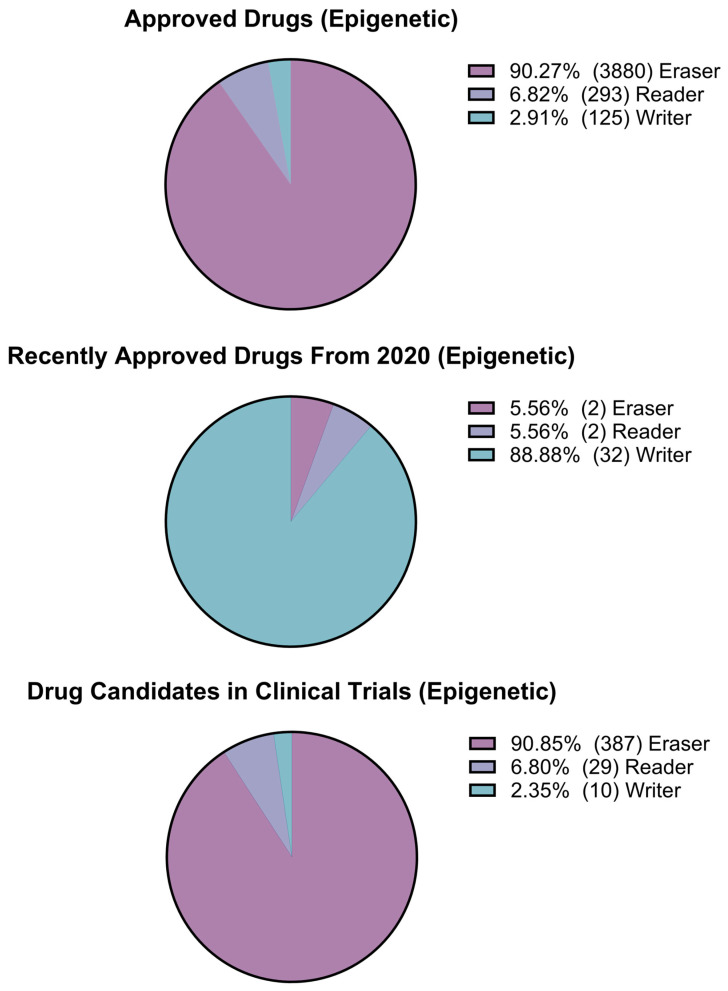
Pharmacological space analysis of epigenetic targets for the Approved Drugs, Approved Drugs after 2020, and Drug Candidates in Clinical Phase datasets.

After collecting pharmacological data from the ChEMBL database, data analysis was performed to generate three sets of graphs. The first set of charts displays the total count of targets for different pharmacological classes, such as enzymes (non-kinase), kinases, GPCRs, ion channels, epigenetic targets, nuclear receptors, structural proteins, transcription factors, and others (Figure 8). A second analysis of enzyme targets was performed considering hydrolases, isomerases, cytochromes, ligases, lyases, NTPases, phosphatases, phosphodiesterases, proteases, reductases, transferases, and aminoacyltransferases, as shown in Figure 9. Kinases were included in the analysis of enzyme targets for comparison (Figure 9). The third analysis focused on epigenetic targets, specifically readers, writers, and erasers, as illustrated in Figure 10.

Based on the data presented in Figure 8, G protein-coupled receptors (GPCRs), which were once considered the primary pharmacological targets [12], are now being surpassed by other target classes, particularly kinases and epigenetic targets. Kinases, which account for 15% of the described activities, represent 17% of newly approved drugs and account for 49% of described activities for small molecules in the clinical phase. In the scenario of drugs on the market, kinases have approximately half the activity data of non-kinase enzymes for drugs on the market. Impressively, in newly approved drugs, the proportion of kinase activity data is more than 50% of all other activities. Ion channels remain widely pursued targets, with a large share of activity data in approved drugs and an even higher share of clinical candidates. The percentage of drugs that target epigenetic modulation has doubled in clinical candidates compared to overall approved drugs.

The comparison between kinases and other enzymes is emphasized in Figure 9, where kinases increase from 41.5% of activity data in approved drugs to 83.8% in newly approved drugs, and 39.3% in the small-molecule clinical phase data set. Figure 10 presents an interesting visualization of the pharmacological space for epigenetic targets. It is noteworthy that erasers, which previously dominated the activity space for approved and newly approved drug targets, no longer account for activity counts for small molecules in the newly approved drugs. In this scenario, writers account for 88.9% of the activities, raising questions about whether there is still room for exploration or interest in the context of erasers.

## 3. Materials and Methods

Chemical space analysis. Chemical data were extracted from the CHEMBLdb34 SQLite format. The data were processed and analyzed in the KNIME program. The MOLECULE_ATC_CLASSIFICATION table was selected, and an inner join was performed with the MOLECULE_DICTIONARY, COMPOUND_PROPERTIES, COMPOUND_STRUCTURES, and ATC_CLASSIFICATION tables. The data were filtered to select approved drugs and clinical candidates with small molecule characteristics, using “max_phase ≤ 4”. “max_phase > 0”. and “mw_freebase < 1000”.

RDKit salt stripper was used to remove salts, keeping only the largest component. Inorganic drugs and drugs with a molecular weight below 100 were then removed. A Vernalis Speedy SMILES Remove s-Block metal-containing molecules filter and Speedy SMILES element count (C, N, O) nodes were used to filter metal-containing molecules and molecules with carbon atom count lower than 2. Smiles strings were used for further duplicate removal after standardization employing RDKit tools based on a Python script (Figure S6, Supplementary Material). RDKit descriptor calculation node was employed to calculate descriptors, such as number of aromatic rings and fraction of sp^3^ carbons.

Fingerprints were calculated based on the RDKit and CDK KNIME nodes, which were used in default settings. Overall, 2048 bits were used for Morgan and FeatMorgan fingeprints. The bit vectors were expanded to generate integer columns. Data were reduced to two dimensions in the umap Python library. The Jaccard metric was employed, and the following settings were used: n_neighbours = 15; n_components = 2; min_dist = 0.1.

The reduced dimensions were then used to perform a clustering calculation using the k-medoids node. The k value was selected based on silhouette coefficient calculation performed on KNIME node. The k-medoids algorithm was also used to select the most representative drugs from each cluster. KNIME nodes such as normalizer, color manager and scatterplot were employed for data visualization.

Pharmacological space analysis. Biological activity data were extracted from the CHEMBLdb34 SQLite format. The data were processed and analyzed in the KNIME program. ACTIVITIES and ASSAYS tables were joined based on assay_id. TARGET DICTIONARY was then joined based on tid (target id) reference. Based on tid reference, this table was joined to an additional table comprising information from PROTEIN_CLASSIFICATION, COMPONENT_CLASS, and TARGET_COMPONENTS. Molregno ID from datasets generated in chemical space analysis (Approved Drugs, Approved Drugs after 2020, and Drug Candidates in Clinical Phase) was used to collect activity data and target annotation from the table generated with biological activities. Extraction was performed, using DB Row Reader node. The generated tables were further filtered for duplicates and threshold of activity < 10 µM and analyzed in KNIME.

## 4. Conclusions

In conclusion, this study provides an overview of the chemical and pharmacological space of drugs, highlighting some trends and patterns. The clustering results obtained reinforce the conclusion that multiple steps of clustering based on chemical fingerprints can help in understanding their hierarchy, showing how subclusters relate to main clusters. Nevertheless, it is challenging to capture all the complexities of chemical space in two dimensions. For example, the analysis of clinical candidates did not yield any meaningful insights, failing to reveal new trends. In contrast, the comparison with drugs approved after 2020 yielded valuable insights. The analysis based on chemical descriptors, such as aromatic ring count and fraction of sp^3^ carbons, suggested which chemotypes are more prevalent in drugs. The shift in cluster density, when comparing approved drugs with recently approved drugs, is corroborated by the clear interest in developing kinase inhibitors, which is highlighted by pharmacological space analysis. Comprehensive pharmacological space analysis reveals shifts in target preferences, with certain classes of targets gaining prominence over others. Specifically, kinases continue to be a major drug target class, confirming a trend that began in the early 2000s. Furthermore, the data indicate that epigenetic writers are an emerging target class. The significance of ongoing exploration of the chemical and pharmacological space to foster innovation in drug discovery underscores the importance of data in maximizing the potential for identifying drug candidates. The methods employed in this work aimed to provide an approach for chemical and pharmacological space study, which may assist future research groups in advancing their drug discovery projects.

## Figures and Tables

**Table 1:** Maximum mean silhouette coefficient for approved drugs based on PubChem-based UMAP embedding.

Cluster Number	Best Silhouette Score for k ≤ 5	Best Silhouette Score for k ≤ 100
Cluster 1	k = 3 0.47	k = 82 0.60
Cluster 2	k = 4 0.51	k = 63 0.52
Cluster 3	k = 2 0.65	k = 33 0.63

**Table 2:** Comparison of the number of approved drugs per cluster when comparing approved drugs and drugs approved after 2020.

Cluster Number	Number of Approved Drugs	Number of Approved Drugs after 2020
Cluster 1	863	16
Cluster 2	625	62
Cluster 3	346	9

## Data Availability

Data are contained within the article.

## Supplementary Materials

The following supporting information can be downloaded at https://www.mdpi.com/article/10.3390/ph17060742/s1. Figure S1. Mean silhouette coefficient for approved drugs based on k-medoids algorithm. Figure S2. Mean silhouette coefficient for Cluster 1 based on k-medoids algorithm. Figure S3. Mean silhouette coefficient for Cluster 2 based on k-medoids algorithm. Figure S4. Mean silhouette coefficient for Cluster 3 based on k-medoids algorithm. Figure S5. Superposition of chemical space of clinical candidates and approved drugs. Clinical candidates are colored in black and approved drugs are colored based on subclusters of Clusters 1, 2, and 3. Figure S6. Python script for smiles standardizing, based on “working_with_ChEMBL_drug_data.ipynb” script available on https://github.com/PatWalters/practical_cheminformatics_tutorials/blob/main/misc/working_with_ChEMBL_drug_data.ipynb (accessed on 2 June 2024).
