# Supplementary material for: Remapping the Chemical Space and the Pharmacological Space of Drugs: What Can We Expect from the Road Ahead?
Source: Pharmaceuticals (Basel). 2024 Jun 6;17(6):742. doi: 10.3390/ph17060742 (PMC11207054; doi:10.3390/ph17060742)
Supplement: Supplementary file 1 [file pharmaceuticals-17-00742-s001.zip › pharmaceuticals-2967625-supplementary.pdf]

Supplementary Material

Remapping the Chemical Space and the Pharmacological Space of Drugs: What Can We Expect from the Road Ahead?

Lucas Silva Franco,[1,2] Bárbara da Silva Mascarenhas de Jesus,[1,3] Pedro de Sena Murteira Pinheiro[1,] and Carlos Alberto Manssour Fraga[1,2,3,*,†]*

1 Laboratório de Avaliação e Síntese de Substâncias Bioativas (LASSBio), Instituto de Ciências Biomédicas, Universidade Federal do Rio de Janeiro, Cidade Universitária , Rio de Janeiro 21941-902, Brazil; silvafrancolucas@gmail.com (L.S.F.); mascarenhas.barbi@gmail.com (B.d.S.M.d.J.)

2 Instituto Nacional de Ciência e Tecnologia de Fármacos e Medicamentos (INCT-INOFAR), , Universidade Federal do Rio de Janeiro, Rio de Janeiro 21941-902, Brazil

3 Programa de Pós-Graduação em Farmacologia e Química Medicinal (PPGFQM), Instituto de Ciências Biomédicas, Universidade Federal do Rio de Janeiro, Cidade Universitária, Rio de Janeiro 21941-902, Brazil

** Correspondence: pedro.pinheiro@icb.ufrj.br (P.d.S.M.P.); cmfraga@ccsdecania.ufrj.br (C.A.M.F.)*

† In memoriam

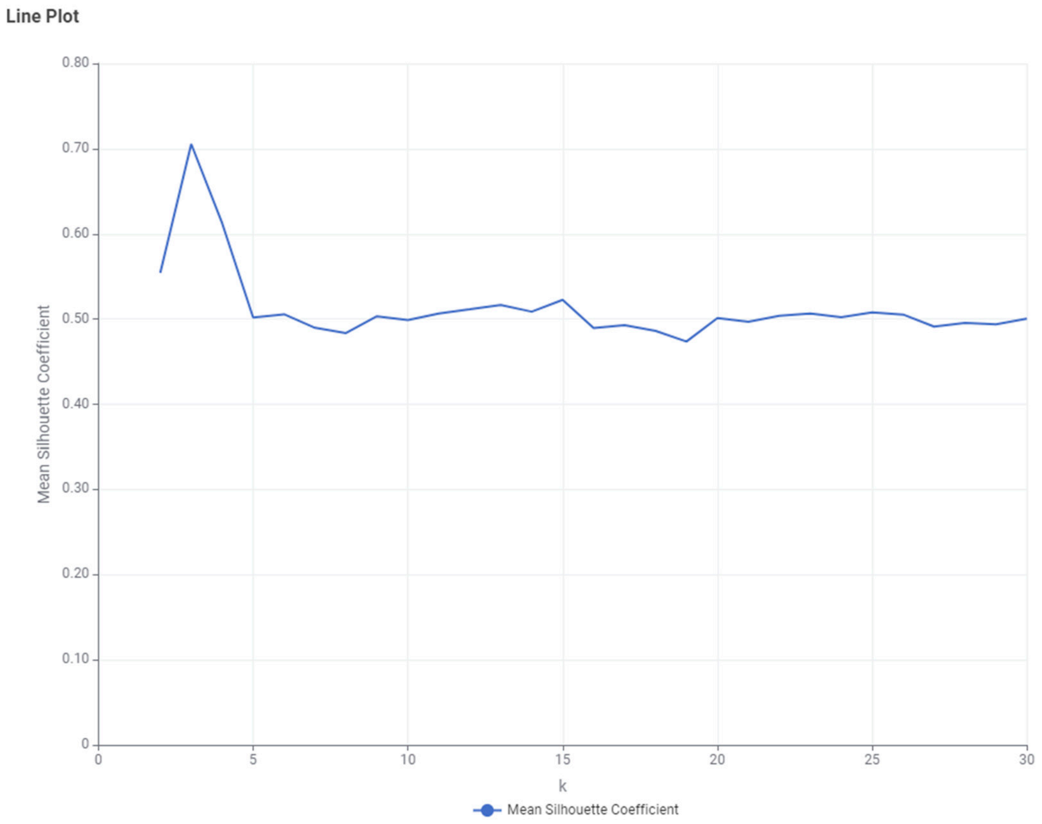

Figure S1. Mean silhouette coefficient for Approved Drugs based on k-medoids algorithm.

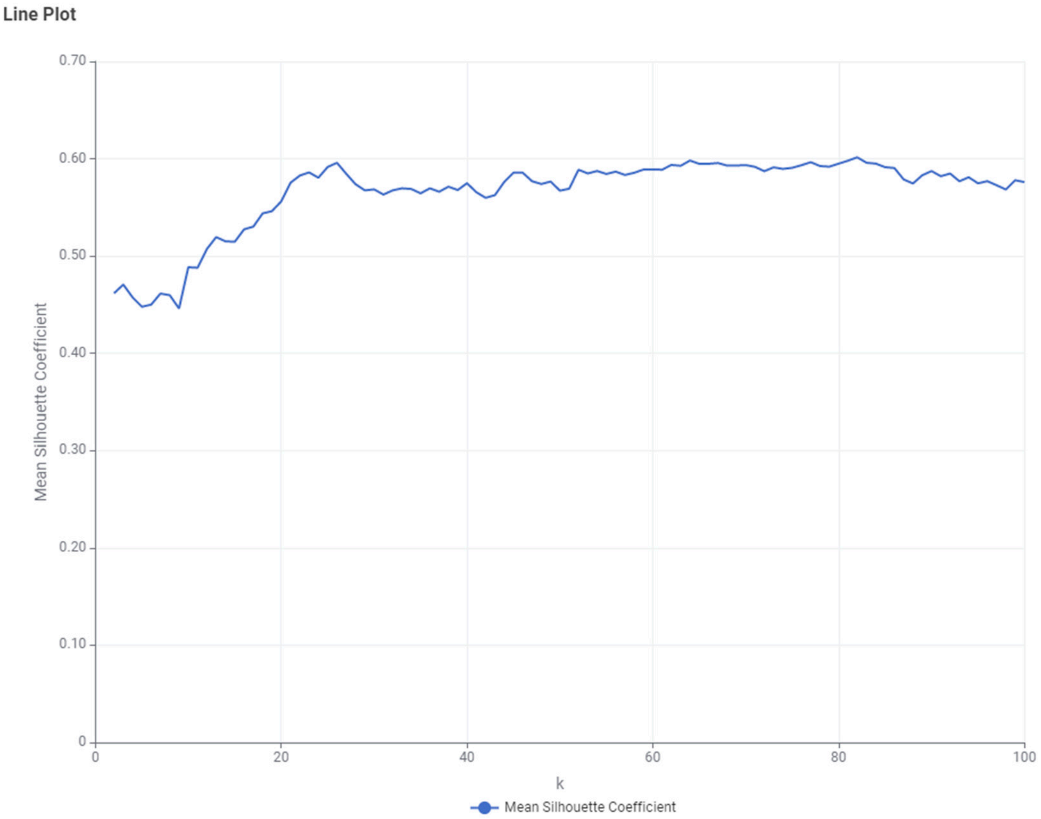

Figure S2. Mean silhouette coefficient for Cluster 1 based on k-medoids algorithm.

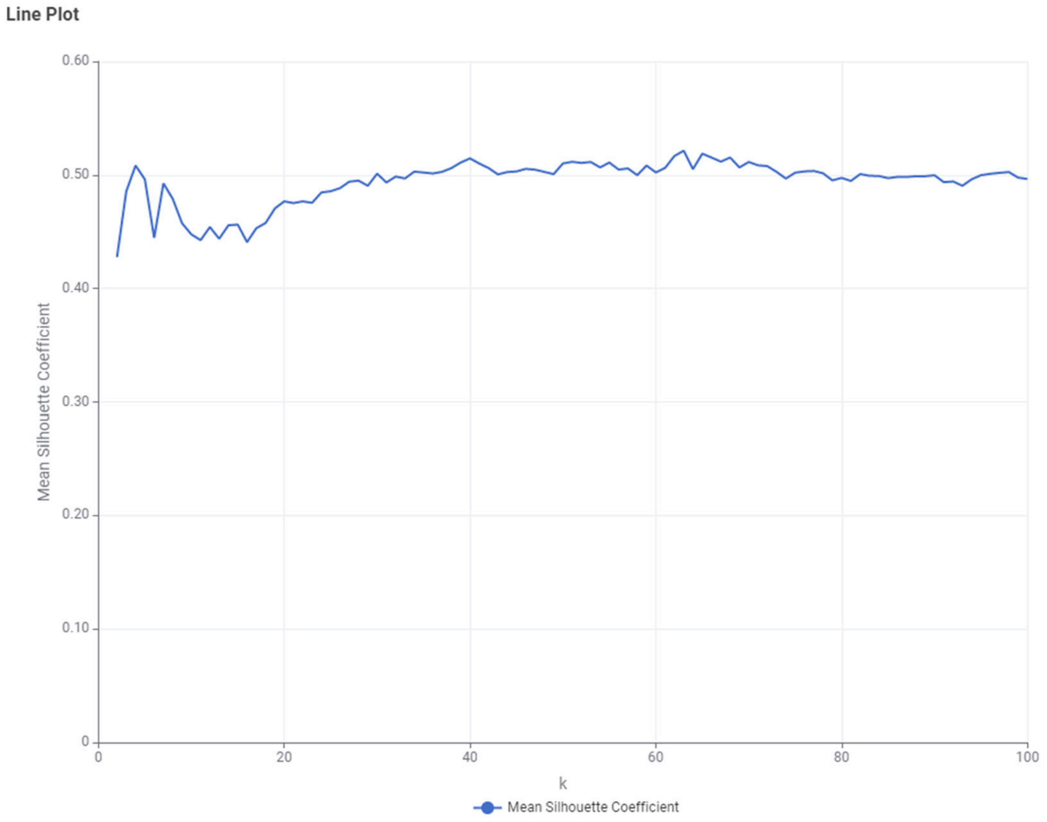

Figure S3. Mean silhouette coefficient for Cluster 2 based on k-medoids algorithm.

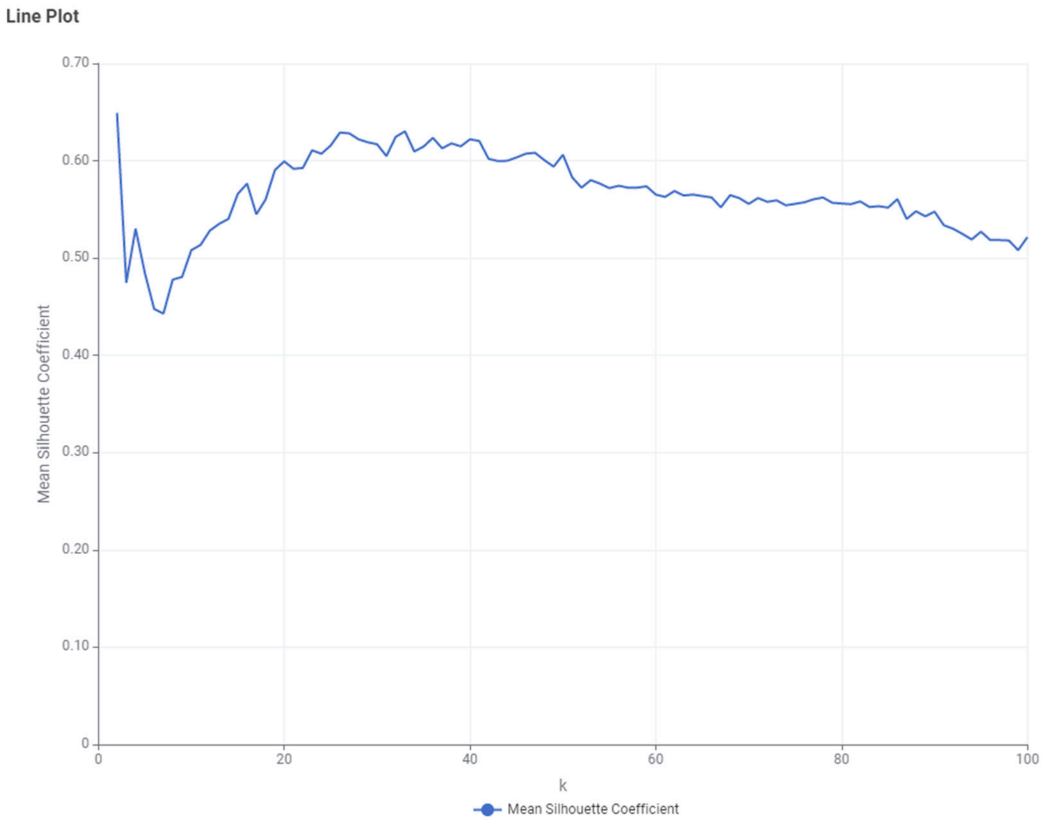

Figure S4. Mean silhouette coefficient for Cluster 3 based on k-medoids algorithm.

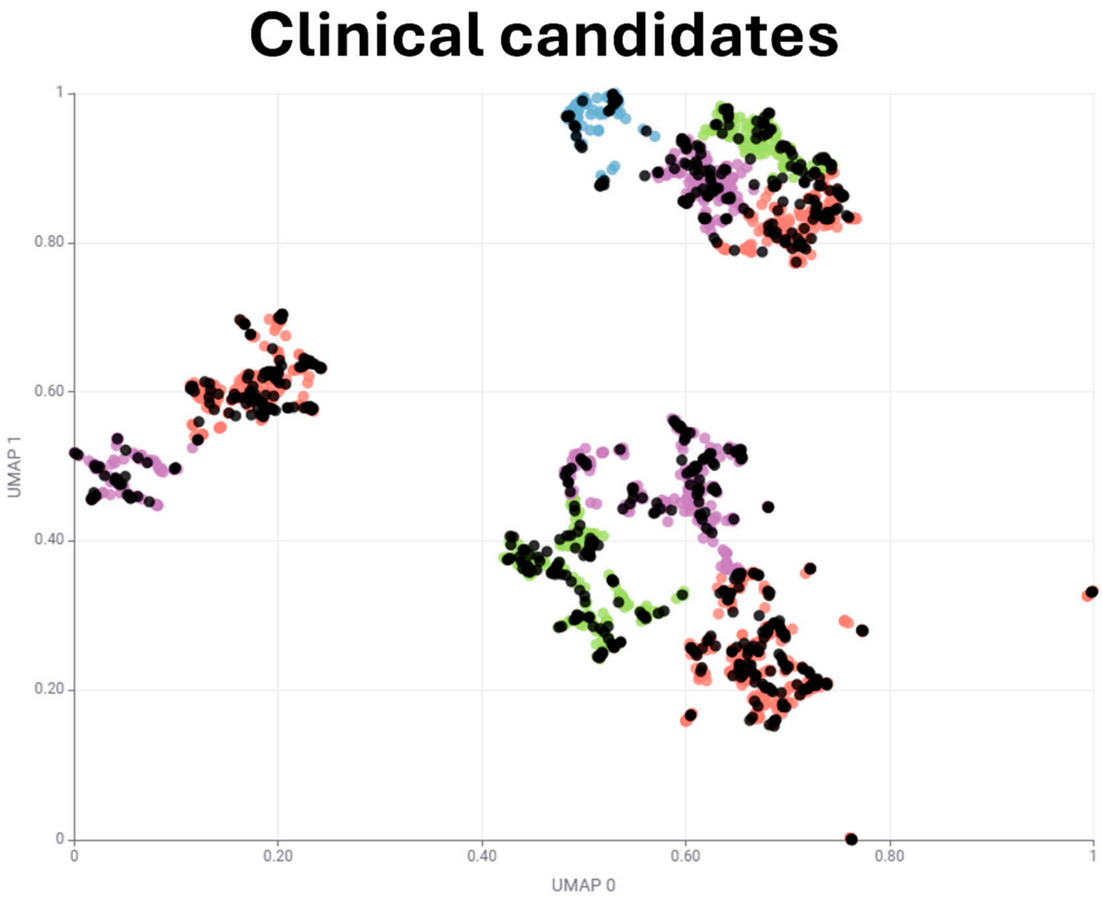

Figure S5. Superposition of chemical space of clinical candidates and approved drugs. Clinical candidates are colored in black and approved drugs are colored based on sub-clusters of Cluster 1, 2 and 3.

```python
import pandas as pd
from rdkit import Chem
from rdkit.Chem.MolStandardize import rdMolStandardize
from tqdm.auto import tqdm

# Function to standardize a molecule from its SMILES string
def standardize(smiles):
  try:
    mol = Chem.MolFromSmiles(smiles)
    if mol is None:
      raise ValueError(f"Invalid SMILES string: {smiles}")

    # Clean up the molecule (removeHs, disconnect metal atoms, normalize, reionize)
    clean_mol = rdMolStandardize.Cleanup(mol)

    # Uncharge the molecule
    uncharger = rdMolStandardize.Uncharger()
    uncharged_parent_clean_mol = uncharger.uncharge(clean_mol)

    # Canonicalize tautomers
    te = rdMolStandardize.TautomerEnumerator()
    taut_uncharged_parent_clean_mol = te.Canonicalize(uncharged_parent_clean_mol)

    return Chem.MolToSmiles(taut_uncharged_parent_clean_mol)
  except Exception as e:
    print(f"Error processing molecule {smiles}: {e}")
    return None

# Read or create the DataFrame
input_table_1 = pd.DataFrame(input_table_1)

# Apply the standardize function to each SMILES string in the DataFrame
tqdm.pandas()  # Progress bar for the apply function
input_table_1['standardized_smiles'] = input_table_1['smiles'].progress_apply(standardize)

# Fill missing values in 'standardized_smiles' with values from 'smiles'
input_table_1['standardized_smiles'] = input_table_1['standardized_smiles'].replace('?',
pd.NA).fillna(input_table_1['smiles'])

# Display the resulting DataFrame
print(input_table_1)
```

Figure S6. Python script for smiles standardizing, based on working_with_ChEMBL_drug_data.ipynb script available on https://github.com/PatWalters/practical_cheminformatics_tutorials/blob/main/misc/working_with_ChEMBL_drug_data.ipynb.
